# CIP4 promotes metastasis in triple-negative breast cancer and is associated with poor patient prognosis

**DOI:** 10.18632/oncotarget.3351

**Published:** 2015-03-19

**Authors:** Otto L.D. Cerqueira, Peter Truesdell, Tomas Baldassarre, Santiago A. Vilella-Arias, Kathleen Watt, Jalna Meens, Harish Chander, Cynthia A.B. Osório, Fernando A. Soares, Eduardo M. Reis, Andrew W.B. Craig

**Affiliations:** ^1^ Departamento de Bioquímica, Instituto de Química, Universidade de São Paulo, São Paulo, SP, Brazil; ^2^ Department of Biomedical and Molecular Sciences, Queen's University, and Division of Cancer Biology & Genetics, Queen's Cancer Research Institute, Kingston, ON, Canada; ^3^ Department of Anatomic Pathology, A.C. Camargo Hospital, São Paulo, SP, Brazil; ^4^ Instituto Nacional de Ciência e Tecnologia em Oncogenômica, São Paulo, SP, Brazil

**Keywords:** metastasis, triple-negative breast cancer, CIP4

## Abstract

Signaling via epidermal growth factor receptor (EGFR) and Src kinase pathways promote triple-negative breast cancer (TNBC) cell invasion and tumor metastasis. Here, we address the role of Cdc42-interacting protein-4 (CIP4) in TNBC metastasis *in vivo*, and profile CIP4 expression in human breast cancer patients. In human TNBC cells, CIP4 knock-down (KD) led to less sustained activation of Erk kinase and impaired cell motility compared to control cells. This correlated with significant defects in 3D invasion of surrounding extracellular matrix by CIP4 KD TNBC cells when grown as spheroid colonies. In mammary orthotopic xenograft assays using both human TNBC cells (MDA-MB-231, HCC 1806) and rat MTLn3 cells, CIP4 silencing had no overt effect on tumor growth, but significantly reduced the incidence of lung metastases in each tumor model. In human invasive breast cancers, high CIP4 levels was significantly associated with high tumor stage, TNBC and HER2 subtypes, and risk of progression to metastatic disease. Together, these results implicate CIP4 in promoting metastasis in TNBCs.

## INTRODUCTION

Breast cancer is a heterogeneous disease with multiple subtypes that differ in risk of relapse [1, 2]. At least four major subtypes have been identified, including luminal A, luminal B, human epidermal growth factor receptor 2 (HER2), and triple-negative breast cancers (TNBC; lacking ER/PR/HER2) [3, 4]. Cluster analysis further subdivides TNBC into basal-like, mesenchymal, mesenchymal stem cell-like, immunomodulatory, and luminal androgen receptor subtypes [4]. TNBC patients frequently progress to metastatic disease [4–6], and since metastasis is a leading cause of cancer deaths [7], improved understanding of metastasis pathways is critical to develop new strategies to treat TNBC patients.

Molecular mechanisms of TNBC cell invasion and tumor metastasis are beginning to emerge, with signaling by EGFR and Src kinases as key players and potential therapeutic targets. Substrates of Src kinase have been implicated in regulation of extracellular matrix (ECM) degradation by invadopodia, cell invasion, and tumor metastasis [8]. A number of actin regulatory proteins act downstream of this Src-dependent pathway [9, 10]. This includes the adaptor protein Cdc42-interacting protein-4 (CIP4), a key regulator of Src signaling and TNBC cell invasion [11, 12]. CIP4 was originally identified as a binding partner of Cdc42 [13], and is targeted to cellular membranes via its F-BAR (Fer/CIP4 homology-Bin1/Amphiphysin/Rvs) domain [14–17]. CIP4 also interacts with proline-rich domains in numerous actin regulatory proteins via its SH3 domain [12, 18–20]. In TNBC cells, CIP4 localizes to invadopodia and promotes N-WASP phosphorylation by Src [12]. Silencing of CIP4 expression in MDA-MB-231 cells resulted in defects in cell motility, invadopodia formation, and cell invasion *in vitro* [12]. In contrast, CIP4 was not required for cell invasion in MDA-MB-231 cells expressing constitutively active Src, and was shown to promote internalization of transmembrane type I matrix metalloproteinase (MT1-MMP) to regulate invadopodia formation [11]. Resolving these differences in phenotypes associated with CIP4 silencing will require further testing in a variety of TNBC cell models and tumor metastasis assays.

Here, we have characterized the role of CIP4 in regulating cell invasion and tumor metastasis in multiple TNBC models using both stable and inducible CIP4 knock-down (KD) approaches. CIP4 KD led to defects in EGFR signaling, cell motility and 3D cell invasion *in vitro*. In mammary orthotopic xenograft assays, CIP4 KD had no overt effect on tumor growth, but impaired metastasis to the lung. Expression profiling of CIP4 in primary tumors from 245 patients with invasive breast carcinoma, has also revealed high CIP4 levels in TNBC and HER2 subtypes, and risk of progression to metastatic disease.

## RESULTS

### CIP4 modulates EGFR signaling in TNBC cells

**Figure 1:**
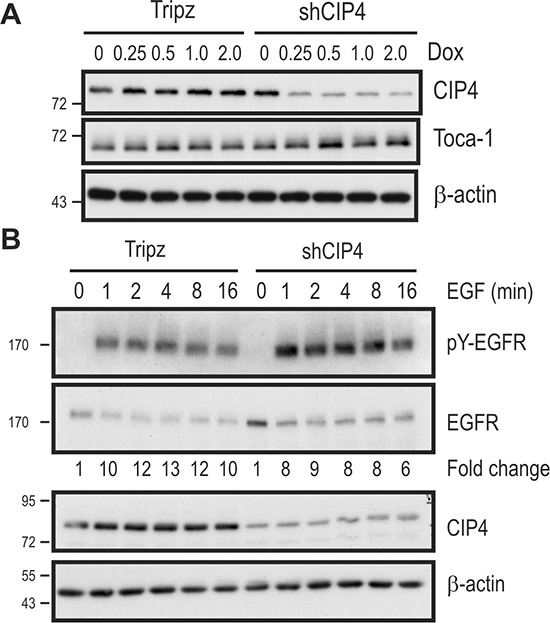
Inducible CIP4 silencing and EGFR activation in TNBC cells alters EGFR signaling **(A)** MDA-MB-231 cells transduced with Tripz (vector) or a Dox-inducible shRNA targeting CIP4 (shCIP4) were treated with the indicated doses of Dox (μg/ml) for 48 hours. Lysates were subjected to immunoblot with the indicated antibodies. **(B)** MDA-MB-231 Tripz or shCIP4 were treated with Dox (2 μg/ml for 48 hours) prior to serum starvation and treatment with EGF (50 ng/ml) for the indicated times (min.). Lysates were subjected to immunoblot with the indicated antibodies. Densitometry was performed and phosphoprotein levels were normalized to total protein levels, and expressed as fold change relative to time 0. Positions of molecular mass markers are indicated on the left.

To better characterize the role of CIP4 in TNBC cells, we used a Tripz-based lentiviral system to allow doxycycline (Dox)-inducible expression of shRNA targeting CIP4 (shCIP4) in several TNBC cell lines. In MDA-MB-231 cells transduced with shCIP4 virus, Dox treatment led to ~80% decrease in CIP4 levels, as measured by immunoblot (Figure 1A). As expected, there was no effect of Dox treatment on CIP4 expression in Tripz vector-transduced cells (Figure 1A). Similar results were also obtained in Dox-treated HCC 1806 cells transduced with both viruses (data not shown). As expected, the Dox-induced silencing of CIP4 had no effect on expression of the related F-BAR adaptor proteins Toca-1 or FBP17 (Figure 1A, data not shown). Since prior studies have implicated CIP4 in regulating EGFR internalization and trafficking to lysosomes for degradation [16, 19], we tested the effects of CIP4 silencing on EGFR levels and signaling in TNBC cells. Consistent with our previous study [16], Dox-induced CIP4 KD led to elevation of EGFR levels at baseline compared to Dox-treated control cells (Figure 1B; second panel). Upon treatment with EGF, robust tyrosine phosphorylation (pY) of EGFR (pY-EGFR) was observed for both cell lines, which indicates normal EGFR activation kinetics in CIP4 KD cells (Figure 1B).

**Figure 2:**
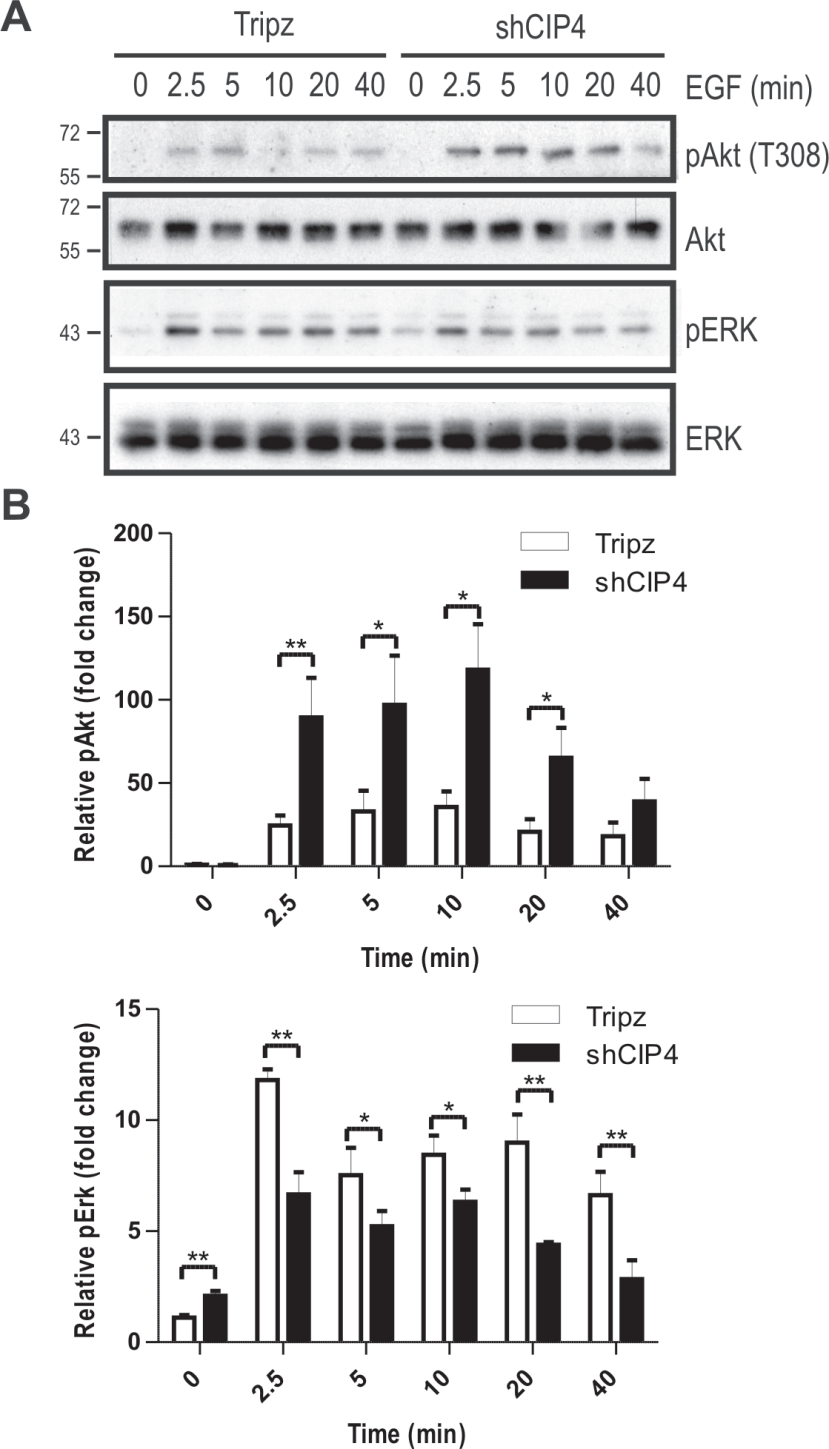
CIP4 silencing alters EGFR signaling to Akt and ERK kinases **(A)** MDA-MB-231 Tripz or shCIP4 were treated with Dox (2 μg/ml for 48 hours) prior to serum starvation and treatment with EGF (50 ng/ml) for the indicated times (min.). Lysates were subjected to immunoblot with the indicated antibodies. Positions of molecular mass markers are indicated on the left. **(B)** Densitometry was performed and phosphoprotein levels were normalized to total protein levels, and expressed as fold change relative to time 0 (mean ± sem is shown for 4 separate blots from 2 experiments; *p < 0.05, **p < 0.01).

To address whether CIP4 regulates EGFR signaling to downstream pathways, we profiled EGF-induced phosphorylation of the activation loop sites (T308) in Akt (pAkt) and Erk kinases (pErk) in Dox-treated vector and CIP4 KD cells. EGF treatment of CIP4 KD cells led to increased pAkt levels compared to control cells (Figure 2A/2B). This may be due to the increased EGFR levels in CIP4 KD cells. Despite elevated EGFR levels in CIP4 KD cells, EGF treatment led to less sustained activation of Erk kinase compared to control cells (Figure 2A/2B). Similar defects in EGFR signaling to Erk and Akt were observed in CIP4 KD lung adenocarcinoma cells [21], and these results implicate CIP4 in regulating EGFR levels and signaling.

### CIP4 promotes TNBC cell invasion

**Figure 3:**
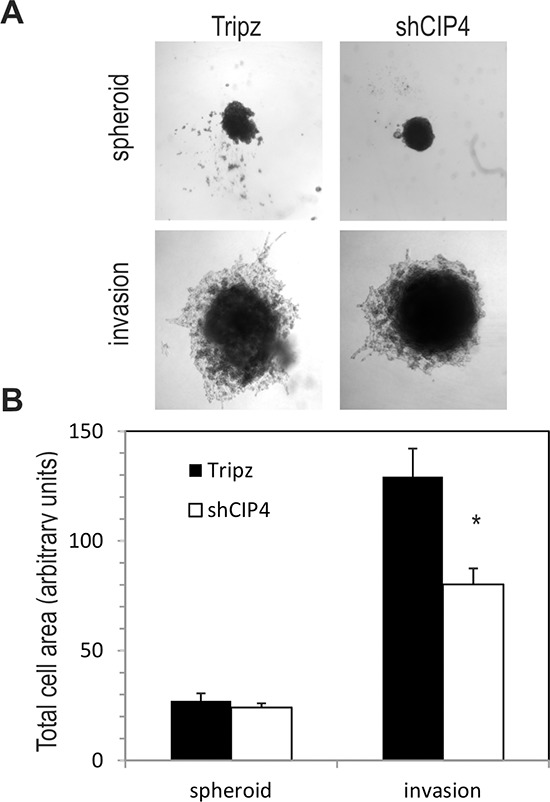
CIP4 promotes TNBC cell invasion **(A)** Representative phase contrast images of spheroid colonies for MDA-MB-231 Tripz or shCIP4 (day 3) and following addition of invasion matrix (day 10, invasion). **(B)** Graph represents total cell area for spheroids colonies and post-invasion colonies (mean ± SD, *p < 0.05; representative results for 1 of 3 experiments).

Next, we tested the effects of CIP4 silencing on TNBC cell motility and invasion through the ECM. Consistent with the previous study by Pichot et al. using transient CIP4 KD [12], we observed a ~50% reduction in cell motility for Dox-treated shCIP4 cells compared to vector control in cell migration assays (Supplementary Figure S1A). The invasive potential of Dox-treated shCIP4 cells was also analyzed using Transwell™ chambers overlayed with Matrigel™, and we observed a ~75% reduction in numbers of invading cells compared to control cells (Supplementary Figure S1B). We also extended these studies to investigate the effects of CIP4 silencing on cell invasion in 3D assays [22]. In conditions of spheroid formation, CIP4 silencing had no effect on cell growth or size of spheroid colonies (Figure 3A). However, upon addition of ECM supplemented with serum, the shCIP4 spheroids remained compact, and showed reduced invasion through the surrounding ECM compared to that of control spheroid colonies (Figure 3A). Quantification of these results from multiple experimental replicates, revealed a significant reduction in 3D cell invasion upon silencing of CIP4 in TNBC cells (Figure 3B). These results are consistent with our recent study of CIP4 in EGFR-driven lung cancer [21], and support a role for CIP4 in promoting TNBC cell invasion.

### CIP4 silencing impairs TNBC tumor metastasis in mice

**Figure 4:**
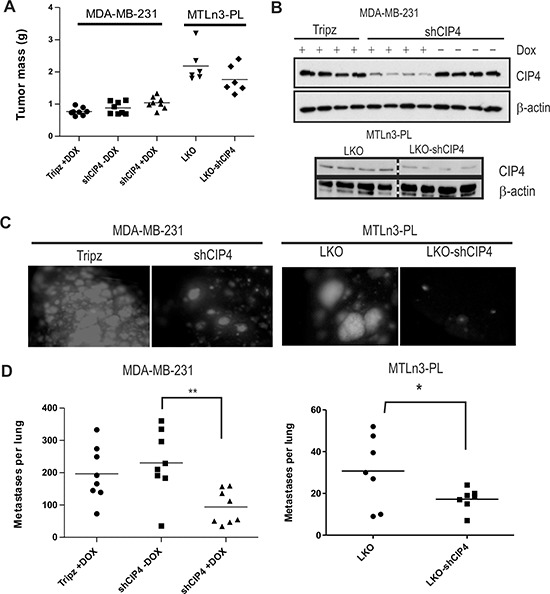
CIP4 promotes TNBC metastasis to the lungs in mice **(A)** Graph depicts primary tumor masses for mammary orthotopic xenograft assays using MDA-MB-231 and MTLn3-PL models (vector or shCIP4). For MDA-MB-231 model, mice were fed either normal chow (shCIP4 –Dox) or Dox-containing chow (Tripz +Dox, shCIP4 +Dox) to regulate shRNA expression *in vivo*. **(B)** Tumor homogenates were subjected to immunoblot with the indicated antibodies to assess the degree of CIP4 silencing *in vivo* for each group (4 tumors were analyzed/group; results are representative of 2 independent experiments). **(C)** Representative images of fluorescent lung metastases for MDA-MB-231 (Tripz +Dox, shCIP4 +Dox; TurboRFP reporter) or MTLn3-PL (LKO, LKO-shCIP4) mammary orthotopic xenograft models. **(D)** Graph represents scoring of lung metastases (per lung) detected in H&E-stained lung tissue sections for each group (*p < 0.05, **p < 0.01).

Next, we investigated whether CIP4 silencing in TNBC cells causes defects in tumor progression and metastasis *in vivo*. Mammary orthotopic xenograft assays were performed using Dox-inducible CIP4 KD in human TNBC cell models (MDA-MB-231 or HCC1806 cells), and by stable silencing of CIP4 in MTLn3 rat mammary adenocarcinoma model, as previously described [23]. To regulate expression of shCIP4 and TurboRFP reporter in the human TNBC models, mice were fed either Dox-supplemented chow or regular chow. This allowed for comparisons of the CIP4 KD group (shCIP4 +Dox) to both a vector control (Tripz +Dox) and an untreated shCIP4 group that lack shRNA expression (shCIP4 –Dox). After 4 weeks the animals were euthanized and primary breast tumors and lung tissues were dissected. We observed no significant differences in the size or mass of primary tumors with CIP4 silencing in MDA-MB-231 or MTLn3 models (Figure 4A). For the HCC 1806 model, tumor sizes were slightly smaller in the shCIP4 groups, but this did not correlate with Dox treatment (Supplementary Figure S2A). To assess the degree of CIP4 silencing *in vivo*, we prepared homogenates from primary tumors and analyzed CIP4 expression levels by immunoblot. In MDA-MB-231 tumors, CIP4 levels were reduced in the shCIP4 +Dox group compared to control groups (shCIP4 –Dox, Tripz +Dox; Figure 4B). CIP4 levels were also reduced in MTLn3 tumors for the shCIP4 group compared to vector control (Figure 4B, lower panels). For the HCC 1806 model, CIP4 levels were low in shCIP4 +Dox, and in some from the –Dox group as well (Supplementary Figure S2B), suggesting some leaky expression of the shRNA in this model. These differences in tumor mass with CIP4 KD may represent differing requirements of CIP4 in basal-like breast tumors (HCC 1806) and mesenchymal-like (MDA-MB-231) TNBC tumor subtypes.

Next, we examined lung tissues for presence of metastases marked by fluorescent reporter genes (Dox-induced TurboRFP reporter for human TNBCs, constitutive GFP in MTLn3-PL). Interestingly, we observed a striking reduction in lung metastases upon CIP4 silencing in both human TNBC and rat MTLn3 tumor models (Figure 4C, Supplementary Figure S2C). To quantify the numbers of metastases in each experimental group, we prepared lung tissue sections and scored the number of metastatic nodules for the entire lung using imaging software. Analyses were conducted on both TNBC xenograft models, and these showed a significant reduction in lung metastases in the CIP4 KD group (shCIP4 +Dox, or LKO-shCIP4) compared to vector controls or shCIP4 mice lacking Dox (Figure 4D, Supplementary Figure S2D). Taken together, these results provide the first direct evidence that CIP4 promotes metastasis in human and rat TNBC cell models *in vivo*.

To distinguish between roles for CIP4 at early or late stages of metastasis, we tested the effects of CIP4 silencing in our TNBC models on lung seeding efficiency following tail vein injections. These assays revealed similar lung seeding efficiency for HCC 1806 cells with or without CIP4 silencing (Supplementary Figure S3A). Similar results were observed in the MTLn3 cell model with stable CIP4 KD compared to vector (pLKO) control (Supplementary Figure S3B). Quantification of these results revealed no significant differences in the numbers of lung metastases with CIP4 silencing in either cell model (Supplementary Figure S3C). These results suggest that defects in metastases in mammary orthotopic xenograft assays arise due to a role of CIP4 in promoting early steps in the process of tumor metastasis.

### Profiling CIP4 expression in human breast tumors reveals association with risk of metastasis in invasive breast carcinoma patients

Based on our findings that CIP4 enhances TNBC metastasis in mouse models, we tested whether CIP4 levels are altered in human breast tumors, and whether this is linked to clinical outcomes or histopathological features. We used a tissue microarray (TMA) containing a collection of 245 cases of breast cancer patients with invasive carcinoma (94% ductal, 6% lobular) that could be assigned to molecular subtypes based on immunohistochemistry (IHC) staining of relevant markers of each subtype [24]. Luminal A tumors (ER^+^ and/or PR^+^HER2^−^) comprised the majority of these cases (68%), followed by basal-like tumors (ER^−^PR^−^HER2^−^EGFR^+^CK5/6^+^) that represented 17% of cases, HER2^+^ (ER^−^PR^−^HER2^+^) at 9% of cases), and luminal B (ER^+^ PR^−^) at 5% of cases. The prevalence of each subtype in our study is comparable to that described for other cohorts of breast patients reported in the literature [25]. Within our cohort, a higher fraction of patients with luminal A subtype remained metastasis-free after diagnosis compared to other subtypes (Supplementary Figure S4A). This is consistent with previous studies of metastasis risk in patient populations [26, 27]. However, the high rates of metastasis within our luminal B subtype was somewhat unexpected (Supplementary Figure S4A), but may be due to the limited numbers of cases in our cohort.

**Figure 5:**
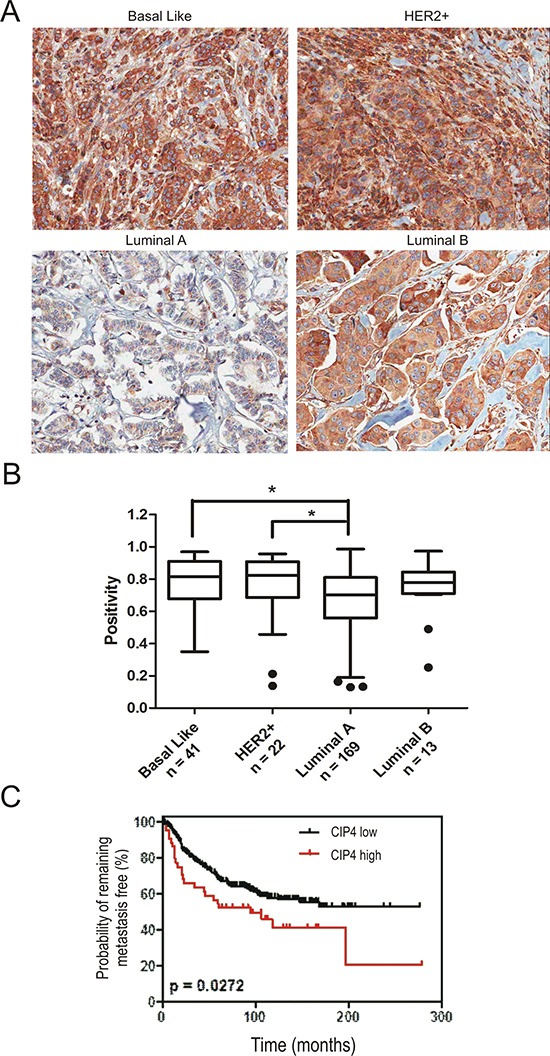
Expression of CIP4 varies between TNBC subtypes and is linked to risk of metastasis **(A)** TMAs containing 245 invasive breast carcinoma patient samples were profiled for CIP4 expression by IHC as described in Materials and methods. Representative images of CIP4 expression in basal-like, HER2, luminal A and luminal B tumors are shown. **(B)** Imaging software was used to score CIP4 levels (positivity), and graph shows CIP4 positivity levels for each subtype (number of cases/subtype indicated below; *indicates a significant difference between luminal A and basal-like or HER2 subtypes, *p* < 0.05). **(C)** Kaplan-Meier graph was prepared to compare risk of developing metastases in patients with high CIP4 (positivity > 0.87) or low CIP4 (positivity < 0.87) levels for the indicated time of follow up (Months). Chi-square test indicated a significant difference between CIP4 high (*N* = 44) and low (*N* = 201) patients (*p* = 0.027).

To address CIP4 expression in primary tumors from this cohort, sections from our TMA were analyzed by IHC staining with a mouse monoclonal antibody raised against human CIP4. The specificity of this antibody was validated using control and CIP4 KD TNBC tumor homogenates from our xenograft assays (Supplementary Figure S4B). In sections with visible normal breast tissue, CIP4 levels were highest in myoepithelial cells compared to alveolar epithelial cells (Supplementary Figure S4C). Within tumor tissues, we observed lowest levels of CIP4 in luminal A tumors compared to basal-like and HER2^+^ subtypes (Figure 5A). Imaging software was used to quantify CIP4 staining (Supplementary Figure S4D), and this revealed significant differences in CIP4 positivity between luminal A and either HER2 or basal-like subtypes (Figure 5B; *p* < 0.05). Intermediate levels of CIP4 were observed in luminal B tumors compared to other subtypes, but these differences were not statistically significant (Figure 5B). Next, we investigated the association between CIP4 levels and clinicopathogical parameters and outcomes within our cohort of invasive breast carcinoma patients with defined molecular subtypes (*n* = 245). First, we determined an optimal positivity value cutoff using the X-Tile software [28], and used this value to group samples with low CIP4 levels (positivity < 0.87; *n* = 201) or high CIP4 levels (positivity > 0.87; *n* = 44). To investigate the association between CIP4 staining and clinicopathological parameters in these breast cancer patients, a univariate correlation analysis was performed using a Chi-square test (Table 1). This analysis revealed statistically significant associations between CIP4 levels and tumor stage (*p* = 0.041), hormone receptor status (*p* < 0.001), HER2 status (*p* = 0.025), and the Perou/Sorlie classification of molecular subtypes (*p* < 0.001). To further assess CIP4 levels and patient outcomes, Kaplan-Meier curves were prepared for the risk of developing metastasis in this cohort of patients according to those with high CIP4 levels or low CIP4 levels (Figure 5C). This analysis revealed a significantly higher probability of patients with high CIP4 levels developing metastases compared to control (Figure 5C, *p* = 0.027). Taken together, our tumor profiling results identify CIP4 as a potential poor prognosis biomarker in human breast cancer patients. Considering our functional data implicating CIP4 in metastasis in multiple breast cancer models (Figure 4), it is likely that high levels of CIP4 expression in human breast tumors correlates with enhanced ability to progress to a metastatic phenotype.

**Table 1:** Correlation of CIP4 expression with clinicopathological and molecular features of human invasive ductal carcinomas

Feature	Total (*n* = 245)	CIP4 IHC staining				*p*-value^b^
		CIP4 low^a^		CIP4 high^a^		
		(*n* = 201)	%	(*n* = 44)	%	
Age						0.315
range 24 – 50	95	75	79	20	21	
range 51 – 92	150	126	84	24	16	
age (mean ± s.d.)		56 ± 0.89		54.8 ± 1.77		
Menopausal status						0.821
Pre	91	74	81	17	19	
Post	154	127	82	27	18	
Tumor Size (cm)						0.118
≤ 2	30	27	90	3	10	
> 2 and ≤ 5	139	117	84	22	16	
> 5	76	57	75	19	25	
Tumor Grade						0.301
1	37	33	89	4	11	
2	141	117	83	24	17	
3	66	51	77	15	23	
n.d.^c^	1					
Tumor Stage						**0.041**
1 + 2	145	125	86	20	14	
3 + 4	100	76	76	24	24	
Lymph node status						0.508
Negative	86	73	85	13	15	
Positive	157	128	82	29	18	
ER						< **0.001**
Negative	71	47	66	24	34	
Positive	174	154	89	20	11	
PR						< **0.001**
Negative	114	82	72	32		28
Positive	131	119	91	12		9
HER2						**0.025**
Negative	210	177	84	33		16
Positive	35	24	69	11		31
Perou/Sorlie classification						< **0.001**
Luminal	182	162	89	20	11	
HER2	22	13	59	9	41	
Basal-Like	41	26	63	15	37	

a CIP4 low: Positivity < 0.87, CIP4 high: Positivity ≥ 0.87; Percentages of samples for each variable are shown in parentheses; values in bold indicate statistically significant correlations (*p* < 0.05); s.d. means standard deviation;

b *p* values to evaluate the dependency between each variable and CIP4 staining were calculated using a Chi-square test;

c n.d. means no data

## DISCUSSION

CIP4 is an F-BAR protein that regulates actin-based cell motility [12, 16, 17, 29]. Prior studies identified differing requirements for CIP4 in regulating invadopodia formation, cell invasion and endocytosis of MT1-MMP in TNBC cells *in vitro* [11, 12]. However, in this study we extend these studies to multiple human and rat models of breast cancer metastasis *in vivo*. We show that inducible silencing of CIP4 results in defects in EGFR signaling, and impaired motility and invasion of TNBC cells. We also tested the effects of CIP4 silencing on TNBC metastasis in tumor xenograft assays, and observed a key role for CIP4 in promoting early steps in TNBC metastasis. Importantly, our study also profiled CIP4 expression in a cohort of human breast cancer patients, which revealed links between high CIP4 levels and risk of developing metastatic disease. Interestingly, another group has recently profiled CIP4 levels in another breast cancer patient cohort, and found similar associations with molecular subtypes and adverse events [30]. In addition, this study provides further evidence for an EMT-promoting role of CIP4 in mammary epithelial cells that is required for motility and cell invasion [30]. Together with our findings in TNBC models, these studies identify CIP4 as a key signaling hub in normal breast epithelial cells and multiple subtypes of breast cancer.

Since the vast majority of basal-like TNBC express high levels of EGFR [4, 31], there has been considerable interest in testing EGFR inhibitors in this subtype that currently lacks targeted therapies [32]. Internalization of EGFR via clathrin-mediated endocytosis involves a number of F-BAR proteins, including CIP4 [19, 33]. In this study, we show that CIP4 KD in TNBC cell lines results in impaired EGFR signaling to ERK MAPKs. Since ERK promotes expression of EMT and MMP genes in TNBC cells [34–36], this may reflect a role for CIP4 in promoting EMT in TNBC, as was recently shown in normal kidney and mammary epithelial cells [30, 37]. We recently showed that CIP4 promotes activation of an EGFR/ERK/Zeb1/MMP-2 axis in lung adenocarcinoma cells and tumors [21], and it will be interesting to further test this axis in CIP4 KD breast tumors. Others have also reported a role for CIP4 in promoting Src activation and Cadherin switching in mammary epithelial cells treated with EGF or TGFβ [30]. Considering that autocrine TGFβ signaling has been implicated in promoting MDA-MB-231 cell invasion [38], we do not discount the potential contributions of multiple pathway defects to explain the cell invasion and tumor metastasis defects we have observed in our CIP4 KD TNBC models.

This study extends our understanding of how early events in breast tumor metastasis are regulated by CIP4. Other recent studies have also implicated CIP4 in promoting metastasis in xenograft models of osteosarcoma and lung cancer [21, 39]. Both our study and the osteosarcoma study, have identified a key role for CIP4 in promoting metastasis. Considering the significant defects in lung metastasis with CIP4 KD in mammary orthotopic xenograft assays, but not in experimental metastasis assays via tail vein injections, this suggests a role for CIP4 in early events in the process of tumor metastasis. This may correspond to a role for CIP4 in EGFR-driven pathways of EMT, invadopodia formation, and cell invasion. Further studies are required to fully understand the defects in CIP4 KD tumor cells during localized invasion of tissues or blood vessels, including the use of intravital microscopy methods [40].

Using a TMA containing 245 cases of invasive breast carcinoma (230 ductal, 15 lobular) we found that luminal A subtype showed lower expression of CIP4 compared to basal-like and HER2 subtypes. Within this cohort, we found that patients with luminal A tumors had reduced risk of developing metastasis compared to patients with basal-like or HER2 subtypes. While these findings are consistent with previous studies [26, 27], this differential expression of CIP4 between subtypes likely accounts for our finding that patients with high CIP4 levels were more at risk for developing metastases. These results were also largely corroborated in an independent cohort of breast cancer patients [30]. However, further testing of CIP4 as a potential poor prognosis biomarker in additional patient cohorts is certainly warranted. In addition, it will be important to extend the tumor profiling studies to include CIP4 expression relative to EGFR levels, and downstream targets that were affected by CIP4 silencing.

In conclusion, our results demonstrate that CIP4 plays a key role in promoting TNBC cell invasion *in vitro*, and tumor metastasis *in vivo*. This implicates CIP4 in both EGFR-driven TNBCs (this paper), and HER2-driven cancer cell invasion [30]. Also, these two studies implicate high levels of CIP4 expression in human breast cancer patients with risk of adverse events [30], or progressing to metastatic disease (this paper). These data implicate CIP4 as a poor prognostic marker in breast cancer, and highlight the importance of further study of this EGFR/CIP4/Erk/MMP-2 signaling axis in other patient cohorts.

## MATERIAL AND METHODS

### Cell lines and reagents

Human TNBC MDA-MB-231 and HCC1806 cell lines were obtained from ATCC. Rat mammary adenocarcinoma MTLn3-PL cells were kindly provided by Jeffrey Segall (Albert Einstein College of Medicine) [41]. Antibodies used in this study included: rabbit anti-CIP4 (used for all immunoblots, described in Ref. [16]), mouse anti-Toca-1 (described in Ref. [16]), mouse anti-CIP4 (used for immunohistochemistry, #sc-166810, Santa Cruz Biotech.(SCBT)), mouse anti-β-actin (C4, SCBT), mouse anti-phospho-EGFR (Y1068, Cell Signaling Tech. (CST)), rabbit anti-EGFR (sc-03, SCBT), rabbit anti-phospho-Akt (T308, C31E5E, CST), rabbit anti-Akt (C67E7, CST), rabbit anti-phospho-p38 (CST), rabbit anti-p38 (CST), mouse anti-phospho-ERK (E-4, SCBT), and rabbit anti-ERK (SCBT). Immunoblots were revealed with HRP-conjugated secondary antibodies (GE Healthcare), and enhanced chemiluminescence reagents (Thermo Scientific). Densitometry was performed on lowest possible exposure autoradiographs using ImageJ software (RSB).

### Stable and inducible silencing of CIP4 knockdown

Lentivirus production and transduction of human and rat TNBC cells was carried out as previously described [23]. For human TNBC cells, pTripz vector and pTripz-shCIP4 (human CIP4 target sequence 5′-CGGCTTTAAACAGCTGGAGAAT-3′; RHS4696–99702419, Open Biosystems) were used to develop Dox-inducible CIP4 KD models. For rat MTLn3-PL cells, pLKO.1 and pLKO.1-shCIP4 (mouse/rat shRNA (C8) target sequence 5′-GTGTGTGGCTATCTACCATTT-3′; TRCN0000173379, Open Biosystems) were used to achieve stable CIP4 KD. MDA-MB-231 and HCC1806 cells were transduced with Tripz or Tripz-shCIP4 lentiviral supernatants (1 ml at 24 and 28 hours) and cell pools were selected using puromycin (2 μg/ml) for 48 hours. The CIP4 KD efficiency was assessed by immunoblotting (IB) with CIP4 antisera on cells treated with Dox (2 μg/ml) for 48 hours. MTLn3-PL cells were transduced pLKO.1 or pLKO.1-C8 (mouse/rat-specific shRNA to CIP4; Open Biosystems) and selected as previously described [23]. Following expansion of cell pools, the efficiency of CIP4 KD in MTLn3-PL cells was assessed by IB with CIP4 antisera.

### Cell migration and invasion assays

Cell migration and invasion assays were performed as described previously [11, 23]. Briefly, TNBC cells were treated with Dox (2 μg/ml) for 48 hours prior to serum starvation followed by plating of 5 × 10^4^ cells in Transwell™ inserts (8 μm pores, Corning; triplicate samples) with or without a layer of Matrigel™ being added (400 μg/cm^2^). The cells were allowed to migrate or invade towards the lower chamber supplemented with 10% FBS for 24 hours, and the numbers of DAPI-stained cells on underside of the filter were quantified using Image-Pro Plus 6 software (Media Cybernetics). Spheroid invasion assays were also performed according to the manufacturer's instructions (Trevigen). Briefly, 3, 000 cells (Tripz or shCIP4) were pretreated with Dox for 48 hours, resuspended in spheroid formation ECM solution, and gently pelleted in a 96 well round bottom spheroid formation plate. After 3 days, spheroids were imaged, and invasion matrix supplemented with 10% FBS was added to each well. The area of each spheroid was measured on day 3 (pre-invasion) and day 10 (post-invasion) using Image-Pro Plus 6 software (Media Cybernetics), and the difference was used to calculate total area of cell invasion.

### Tumor xenograft assays

Mammary orthotopic xenograft assays were conducted in Rag2^−/−^:IL2Rγ_c_^−/−^ mice as previously described [23, 42]. For MDA-MB-231 and HCC1806 cell lines, 1.5 × 10^6^ cells were adjusted to a final volume of 50 μl in 50% Matrigel™ and inoculated using a hypodermic syringe. For xenograft assays using MTLn3-PL LKO and LKO-shCIP4, 5 × 10^5^ cells were injected as above. For studies with Tripz or Tripz-shCIP4 cells, mice were fed either regular chow or Dox-containing chow (625 mg/kg, Harlan Laboratories). After 4 weeks, the animals were sacrificed and the primary tumors were removed and weighed. The lungs were also dissected and imaged using an epi-fluorescence microscope (4X objective) to visualize TurboRFP^+^ metastatic nodules. Primary tumors and lung tissues were formalin fixed and embedded in paraffin. Tissue sections (5 μm) were stained with hematoxylin/eosin (H&E) for histological examination. Scoring of lung metastases was conducted in a blinded fashion using ImageScope software (Aperio). Experimental metastasis assays were also performed using tail vein injections for cells prepared as above in Rag2^−/−^:IL2Rγ_c_^−/−^ mice. After 2 weeks, animals were sacrificed and lungs were subjected to similar analyses of the lungs. All experiments were approved by the Queen's University Animal Care Committee in accordance with Canadian Council for Animal Care regulations.

### Breast tissue microarray and immunohistochemistry

Tissue microarrays (TMAs) were assembled from archival invasive breast carcinoma primary tumor blocks (Hospital A.C. Camargo, São Paulo, Brazil; samples from 1976–2005; clinical follow-up until 2008). For each sample, source tissue blocks were sampled in a representative area of the tumor (TMArrayer punch MP10–1.0 mm) and transferred to a master block using Beecher Tissue Microarrayer Instrument (Beecher Instruments, Silver Spring, MD). Cores from patients that received neo-adjuvant chemotherapy, displaying only in situ lesions, with < 10% tumor area, with < 7 years follow up, and those lacking a defined molecular subtype based on immunohistochemistry (IHC) staining with ER, PR, HER2, EGFR, and cytokeratin (CK5/6) antibodies were excluded (*N* = 245). TMA sections were deparaffinized, rehydrated, and antigen recovery performed in Tris/EDTA pH 9.0 (100ºC for 15 min). Endogenous peroxidase activity was blocked by hydrogen peroxidase treatment. The TMA slides were incubated with mouse anti-CIP4 (#sc-166810, SCBT) at 1:200 in blocking solution for 2 h at room temperature, rinsed with PBS and detected with secondary antibody (Advance TM HRP link – Dako) for 30 min at room temperature and HRP-coupled dextran polymer detection system (Advance HRP link - Dako) for 30 min at room temperature. Following washes with PBS, signals were detected with 3, 3´-diaminobenzine tetrachloride (Liquid DAB + substrate chromogen system; Dako). Omitting the primary antibody resulted in a lack of background staining. TMA slides were counter-stained with hematoxylin, examined under a light microscope (Zeiss), and scanned with a ScanScope AT Turbo image capture system (Aperio ePathology Solutions Inc). DAB staining intensities (“Positivity” values) were calculated using the “Pixel Count V9” algorithm (Aperio ePathology Solutions Inc). In short, the algorithm calculates the positivity value for each spot as the total number of positive pixels divided by the total number of pixels in each region of interest. Scoring was validated by a trained medical pathologist.

### IHC and statistical analyses

Statistical analyses were performed using Graph Pad Prism 5.0 (Graph Pad Software Inc., USA) and Mini-Tab software (v.16). Chi-square test was used in the cross-tabulations analysis to evaluate significant associations between CIP4 positivity and clinicopathological variables. Univariate Kaplan-Meier (KM) survival curves were used to estimate the probability of patients to remain metastasis-free, considering a 300-month follow-up time following surgery. The CIP4 positivity cutoff value applied in the KM analysis was defined by the X-Tile algorithm, which uses a training validation approach to define optimal prognostic cutoffs from continuous tumor biomarker scoring data [28]. In brief, using a randomly selected sample subset (training set), the algorithm first identified the positivity cutoff that best stratified samples in the training according to their probability of remaining metastasis-free. Next, this cutoff was tested in the remaining samples (validation set). This procedure was repeated several times to generate a distribution of cutoff values and highlight the optimal CIP4 positivity cutoff value that best discriminated subpopulations of patients based on the metastasis outcome. Log-rank test was used to assess the statistical significance of KM curves. A *p* ≤ 0.05 significance threshold was applied in all statistical analyses.

## SUPPLEMENTARY FIGURES


