# Supplementary material for: CIP4 promotes metastasis in triple-negative breast cancer and is associated with poor patient prognosis
Source: Oncotarget. 2015 Mar 19;6(11):9397–408. doi: 10.18632/oncotarget.3351 (PMC4496225; doi:10.18632/oncotarget.3351)
Supplement: Supplementary file 1 [file oncotarget-06-9397-s001.pdf]

SUPPLEMENTARY FIGURES

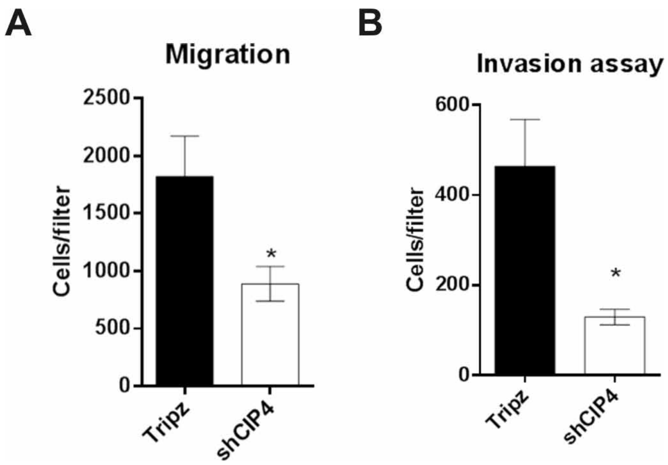

Supplementary Figure S1: CIP4 promotes TNBC cell migration and invasion. (A) MDA-MB-231 Tripz or shCIP4 were treated with Dox (2 µg/ml for 48 hours) prior to performing Transwell migration assays as described in Materials and methods. Total migrating cells per filter were scored for triplicate filters per cell line, and graph represents mean ± SD (*indicates a significant difference between cell lines, $p < 0.05$; representative results for 1 of 3 experiments). **(B)** Cells were treated with Dox as described above and subjected to Transwell invasion assays as described in Materials and methods. Total invading cells per filter were scored for triplicate filters per cell line, and graph represents mean ± SD (*$p < 0.05$; representative results for 1 of 3 experiments).

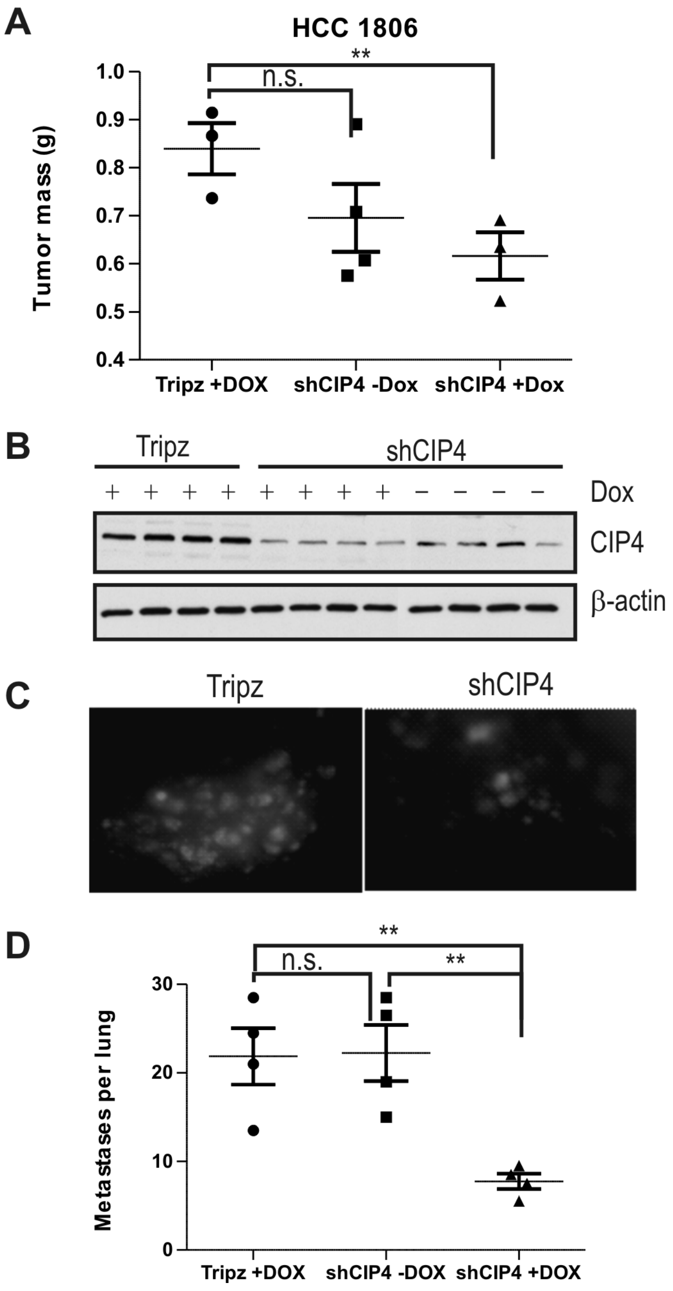

Supplementary Figure S2: CIP4 promotes basal-like breast cancer metastasis in mice. **(A)** Graph depicts primary tumor masses for mammary orthotopic xenograft assays using HCC 1806 (vector or shCIP4). Mice were fed either normal chow (shCIP4 –Dox) or Dox-containing chow (Tripz +Dox, shCIP4 +Dox) to regulate shRNA expression *in vivo* (n.s., not significant; $**p < 0.01$) **(B)** Tumor homogenates were subjected to immunoblot with the indicated antibodies to assess the degree of CIP4 silencing *in vivo* for each group (4 tumors were analyzed/group; results are representative of 2 independent experiments). **(C)** Representative images of RFP+ lung metastases for HCC 1806 Tripz +Dox and shCIP4 +Dox groups from mammary orthotopic xenograft models. **(D)** Graph represents scoring of lung metastases (per lung) detected in H&E-stained lung tissue sections for each group ($**p < 0.01$).

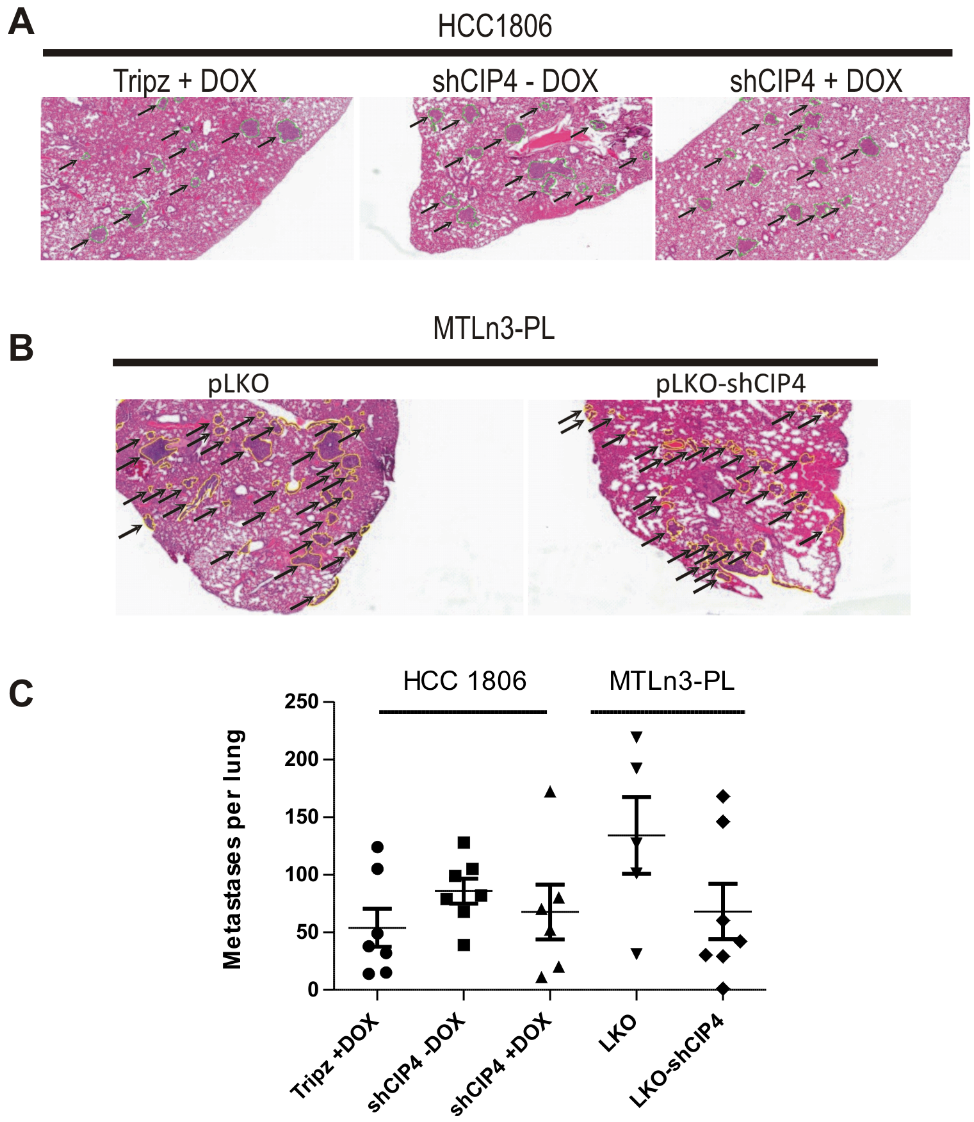

Supplementary Figure S3: CIP4 is not required for lung seeding by breast cancer cells in experimental metastasis assays. **(A)** HCC1806 Tripz and shCIP4 cells were treated with Dox (2 μg/ml; 48 hr) prior to injection in the tail vein of female Rag2$^{-/-}$:IL2Rγ$^{-/-}$ mice. Mice were fed either regular chow (shCIP4 -Dox) or Dox-containing chow (Tripz +Dox, shCIP4 +Dox). At two weeks post injection, mice were sacrificed and lung tissue sections prepared for H&E staining. Representative images are shown with arrows indicating metastatic nodules. **(B)** MTLn3-PL cells transduced with LKO or LKO-shCIP4 were injected in the tail vein of female Rag2$^{-/-}$:IL2Rγ$^{-/-}$ mice. At two weeks post injection, mice were sacrificed and lung tissue sections prepared for H&E staining. Representative images are shown with arrows indicating metastatic nodules. **(C)** Graph depicts the scoring of lung metastases per lung for both cancer cell models (no significant differences were observed).

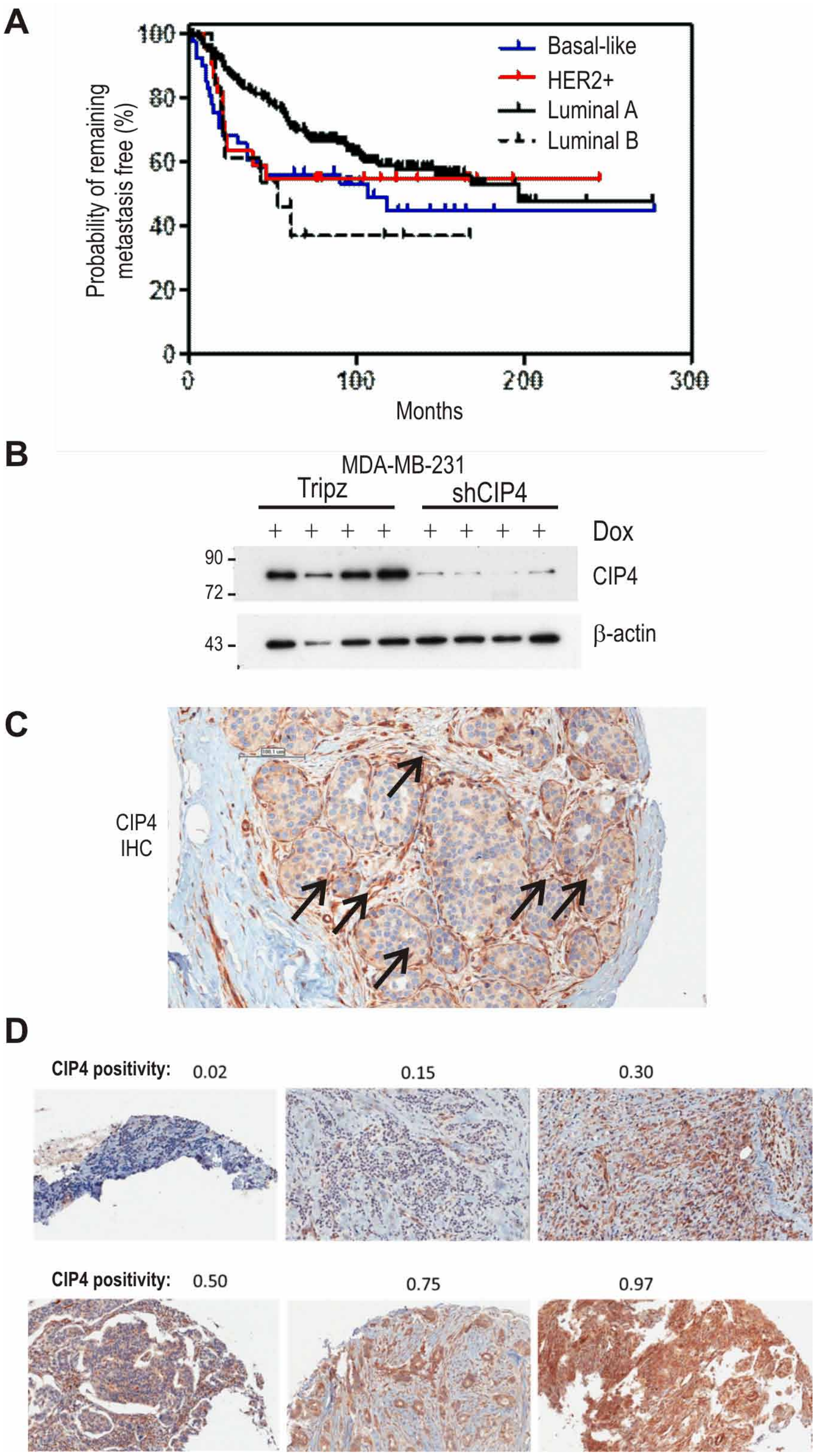

Supplementary Figure S4: Metastasis risk in breast cancer cohort and quantification of CIP4 IHC staining. **(A)** Kaplan-Meier graph depicts the incidence of metastasis in our breast cancer cohort according to molecular subtypes, as described in Materials and Methods. **(B)** Specificity of CIP4 monoclonal antibody was assessed by immunoblot of MDA-MB-231 (Tripz or shCIP4, Dox-treated) tumor homogenates from our mammary orthotopic xenograft assays (β-actin served as a loading control). **(C)** Representative image of CIP4 IHC staining in normal human breast tissue, with high CIP4 expression detected in myoepithelial cells (indicated by arrows) compared to alveolar epithelial cells or stromal cells. **(D)** Representative images of CIP4 IHC staining in human breast tumor samples corresponding to the indicated values of CIP4 positivity (calculated by Pixel count V9, Aperio ePathology Solutions Inc).
